# LAMP-2 deficiency leads to hippocampal dysfunction but normal clearance of neuronal substrates of chaperone-mediated autophagy in a mouse model for Danon disease

**DOI:** 10.1186/s40478-014-0182-y

**Published:** 2015-01-31

**Authors:** Michelle Rothaug, Stijn Stroobants, Michaela Schweizer, Judith Peters, Friederike Zunke, Mirka Allerding, Rudi D’Hooge, Paul Saftig, Judith Blanz

**Affiliations:** Institute of Biochemistry, Christian-Albrechts-Universität zu Kiel, Olshausenstrasse 40, D-24098 Kiel, Germany; Laboratory of Biological Psychology, University of Leuven, B-3000 Leuven, Belgium; Department of Electron Microscopy, Center for Molecular Neurobiology, University Medical Center Hamburg-Eppendorf, D-20246 Hamburg, Germany

**Keywords:** LAMP-2, Danon disease, Mouse model, Lysosome, Chaperone-mediated autophagy, Huntingtin, α-synuclein

## Abstract

The Lysosomal Associated Membrane Protein type-2 (LAMP-2) is an abundant lysosomal membrane protein with an important role in immunity, macroautophagy (MA) and chaperone-mediated autophagy (CMA). Mutations within the *Lamp2* gene cause Danon disease, an X-linked lysosomal storage disorder characterized by (cardio)myopathy and intellectual dysfunction. The pathological hallmark of this disease is an accumulation of glycogen and autophagic vacuoles in cardiac and skeletal muscle that, along with the myopathy, is also present in LAMP-2-deficient mice. Intellectual dysfunction observed in the human disease suggests a pivotal role of LAMP-2 within brain. LAMP-2A, one specific LAMP-2 isoform, was proposed to be important for the lysosomal degradation of selective proteins involved in neurodegenerative diseases such as Huntington’s and Parkinson’s disease.

To elucidate the neuronal function of LAMP-2 we analyzed knockout mice for neuropathological changes, MA and steady-state levels of CMA substrates. The absence of LAMP-2 in murine brain led to inflammation and abnormal behavior, including motor deficits and impaired learning. The latter abnormality points to hippocampal dysfunction caused by altered lysosomal activity, distinct accumulation of p62-positive aggregates, autophagic vacuoles and lipid storage within hippocampal neurons and their presynaptic terminals. The absence of LAMP-2 did not apparently affect MA or steady-state levels of selected CMA substrates in brain or neuroblastoma cells under physiological and prolonged starvation conditions.

Our data contribute to the understanding of intellectual dysfunction observed in Danon disease patients and highlight the role of LAMP-2 within the central nervous system, particularly the hippocampus.

**Electronic supplementary material:**

The online version of this article (doi:10.1186/s40478-014-0182-y) contains supplementary material, which is available to authorized users.

## Introduction

The Lysosomal Associated Membrane Protein type-2 (LAMP-2) is a heavily glycosylated protein that, along with LAMP-1, constitutes the majority of all membrane proteins in the lysosome. The carbohydrates of the LAMP proteins form a glycocalyx lining the inner leaflet of the lysosomal membrane that has a compact appearance of only 8 nm in thickness [1,2]. It is suggested to maintain the structural integrity of the lysosomal membrane. However, deglycosylation of the LAMP proteins reduces their stability but does not affect lysosomal integrity and its degradation capacity [3]. LAMP-2, most likely in concert with LAMP-1, has been proposed to contribute to the maturation of autophagic vacuoles [4,5] and phagosomes [6-8] by promoting vesicular fusion events along microtubules [8] and is also involved in endosomal/lysosomal cholesterol trafficking [9,10].

Alternative splicing of the *Lamp*2 gene produces three isoforms, namely LAMP-2A, LAMP-2B and LAMP-2C that are expressed in a tissue specific manner [11-13]. One particular function of the isoform LAMP-2A is to facilitate the selective import and degradation of cytosolic proteins in the lysosome via chaperone-mediated autophagy (CMA) [14,15] through recognition of a CMA-targeting motif, a pentapeptide sequence biochemically related to KFERQ present within 25–30% of all cytosolic proteins [16].

This isoform is also implicated in major histocompatibility complex class II presentation of cytoplasmic antigens [17] as well as in the regulation of T-cell responses [18]. LAMP-2A is also the rate-limiting factor for the neuronal uptake and degradation of aggregation prone proteins via CMA such as α-synuclein (α-syn) [19,20] and huntingtin (Htt) [21,22] that are neurotoxic when aggregated [23,24]. Mutations within the *Lamp2* gene cause Danon disease, an X-linked “lysosomal glycogen storage disease with normal acid maltase”. Danon disease patients suffer from severe skeletal and cardiac myopathy as well as intellectual dysfunction [25-27]. Interestingly, one Danon disease patient was identified to carry a mutation only affecting LAMP-2B, underlining the importance of this specific isoform [26].

In addition to increased mortality up to post-natal day 40 and reduced size, we have previously described (cardio)myopathy and the pronounced accumulation of autophagic vesicles in cardiac and skeletal muscle of LAMP-2 knockout mice [4,28] similar to those reported in human patients [26]. Neuropathological changes in post-mortem material from a Danon disease patient have been observed [29] which warranted a more in-depth analysis of LAMP-2-deficient murine brain for the presence of neuropathological signs.

Here, we report that absence of LAMP-2 in mice leads to inflammatory changes and lysosomal accumulation of electron dense material within neurons of the central nervous system (CNS). Behavioral abnormalities such as impaired memory point to hippocampal dysfunction caused by perturbed lysosomal activity, accumulation of p62-positive aggregates as well as cholesterol storage within neurons of the hippocampus. In addition, hippocampal neurons displayed a distinct accumulation of lipofuscin and autophagic vacuoles containing amorphous and multilamellar material. Despite its proposed role for the lysosomal degradation of α-syn, Htt, the myocyte-specific enhancer factor-2D (MEF2D) and glyceraldehyde-3-phosphate dehydrogenase (GAPDH) in lysosomes via CMA, steady-state levels of these proteins were unchanged in LAMP-2-deficient brain tissue and in neuroblastoma cells where LAMP-2 was stably down-regulated. Our data demonstrate an essential role of the LAMP-2 protein within the CNS for cognitive functions and autophagy.

## Materials and methods

### Experimental animals

LAMP-2-deficient mice were described previously [4,30]. Only male animals were used in this study due to the X-chromosomal location of the *Lamp2* gene. Therefore, LAMP-2-deficient animals that were backcrossed into C57/BL6-N (Charles River) are referred to as LAMP-2^-/y^. Animals were maintained in a conventional animal facility. All procedures performed in this study involving animals were in accordance with the ethical standards set by the National Animal Care Committee of Germany.

### Materials

Substrates for activity assays (p-nitrophenyl-α-D-glucuronide and p-nitrophenyl-N-acetyl-β-D-glucosiminide) and filipin complex from *Streptomyces filipinensis* were purchased from Sigma Aldrich (Steinheim, Germany). Reagents for molecular biology and protein standards were obtained from Fermentas (St. Leon-Rot, Germany). Chemicals for buffers and RNA isolation were from Roth (Karlsruhe, Germany) and the BCA protein assay kit and Western blotting reagents from Pierce (Rockford, USA) and Amersham (Little Chalfont, United Kingdom), respectively. Complete® protease inhibitor and PefaBloc® were purchased from Roche (Mannheim, Germany). Media for common cell culture was acquired from PAA (Pasching, Austria).

### (Immuno)histology, filipin staining and electron microscopy

For antibody labelling, mice were perfused transcardially with 4% paraformaldehyde (PFA) in 0.1 M phosphate buffer (PB). The brains were dissected and post-fixed for 4 hours. Thereafter, they were embedded in paraffin or incubated in 30% sucrose/0.1 M PB. Five μm paraffin sections or 35 μm free-floating cryosections were cut. 3,3’-Diaminobenzidine (DAB) staining was carried out on free-floating cryosections using the ABC kit followed by the Elite DAB staining kit according to the manufacturer’s instructions (Vector Laboratories, Enzo Life Sciences, Lörrach, Germany). Periodic-Acid-Schiff (PAS) staining was carried out according to common lab protocols. TUNEL staining was performed using the ApopTag® peroxidase *in situ* apoptosis detection kit (Millipore, Schwalbach, Germany) according to the manufacturer´s instructions. Sections were mounted in Eukitt (Sigma Aldrich, Steinheim, Germany) and visualized using a BX50 microscope (Olympus, Hamburg, Germany). Filipin histochemistry was performed on 35 μm vibratome sections at room temperature. Sections were washed 2 × 10 minutes in phosphate buffered saline (PBS), 2 × 10 minutes in 0.2% saponin/PBS and then incubated in filipin (0.05 mg/ml) for 20 minutes. After washing 2 × 10 minutes in 0.02% saponin/PBS and 2 × 10 minutes in PBS, sections were mounted in Prolong anti-fade mounting solution (Invitrogen, Darmstadt, Germany) and stored at 4°C. For electron microscopy (EM) mice were perfused and post-fixed with 4% PFA/1% glutaraldehyde in 0.1 M PB. Brains were dissected and stored in fresh EM fixative at 4°C until further treatment. Following osmication with 1% osmiumtetroxide in cacodylate buffer the sections were dehydrated using ascending alcohol concentration steps, followed by two rinses in propylene oxide. Infiltration of the embedding medium was performed by immersing the pieces in a 1:1 mixture of propylene oxide and Epon (Carl Roth GmbH & Co. KG, Karlsruhe, Germany) and finally in neat Epon before hardening at 60°C for 48 hours. Semithin sections (0.5 μm) were prepared for light microscopy, mounted on glass slides and stained for 1 minute with 1% toluidine blue. Ultrathin sections (60 nm) were cut and mounted on copper grids. Sections were stained using uranyl acetate and lead citrate. Thin sections were examined and photographed using an EM902 (Zeiss, Jena Germany) electron microscope equipped with a Megaview III digital camera (Albert Tröndle, Moorenweis, Germany).

### LAMP-2 knockdown in N2a cells

Oligonucleotides were purchased from Sigma Aldrich (Steinheim, Germany) and siRNA sequences were determined using the DSIR program (http://biodev.extra.cea.fr/DSIR/DSIR.html). The corresponding scramble RNA sequence was created using siRNA wizard (http://www.sirnawizard.com/scrambled.php). Oligonucleotide sequences used were as follows: forward primer LAMP-2 shRNA 5’-3’ [gatccccGGAGATGAATTTCACAATAttcaagagaTATGTGAAATTCATCTCCttttta], reverse primer LAMP-2 shRNA 5’-3’ [agcttaaaaaGGAGATGAATTTCACAATAtctcttgaaTATTGTGAAATTCATCTCCggg], forward primer scmbl shRNA 5’-3’ [gatccccGATTGCTAAGACAAGTAAttcaagagaTTACTTGTCTATAGCAATCttttta], reverse primer scmbl shRNA 5’-3’ [agcttaaaaaGATTGCTATAGACAAGTAAtctcttgaaTTACTTGTCTATAGCAATCggg]. Short hairpin sequences were transferred between the BglII und HindIII sites in the pSUPER vector (OligoEngine, Seattle, USA) according to the manufacturer’s instructions. Mouse neuroblastoma cells (N2a) were transfected using polyethylenimine [31]. Stable transfection with G418 (PAA, Pasching, Austria) was initiated 48 hours post-transfection at a concentration of 100 μg/ml and increased by 100 μg/ml every 48 hours until a maximum of 400 μg/ml G418 was reached. Cells were maintained for at least 2 weeks in stable media before preparation for immunoblotting. Cells were harvested in ice cold PBS with protease inhibitors (complete® from Roche, Mannheim, Germany). Messenger RNA was isolated from HeLa cells using the NucleoSpin® RNAII kit (Macherey-Nagel, Düren, Germany) according to the manufacturer’s instructions. Subsequently, LAMP-2A was cloned in to the pcDNA3.1 Hygro^+^ vector (Invitrogen, Darmstadt, Germany) from cDNA transcribed using the RevertAid cDNA synthesis Kit (Fermentas, St. Leon-Rot, Germany).

### Behavioral study

LAMP-2 knockout mice and wild-type controls (both: n = 4) were examined in a battery of behavioral tests to assess functional consequences of LAMP-2 deficiency. Different tests of motor performance were included. Home cage activity was analyzed in 20 cm vs 30 cm transparent cages which were placed between 3 infrared beams. Total number of beam crossings was recorded for 23 hours during 30 minute intervals (start 4 pm). Grip strength was measured using a T-shaped bar connected to a digital dynamometer (Ugo Basile, Comerio, Italy). Mice were placed in such a way that they grabbed the bar spontaneously and were softly pulled backwards by the tail until they released their grip. Ten such readouts were recorded. Motor coordination and equilibrium were tested using an accelerating rotarod (MED Associates Inc., St. Albans, Vermont, USA). Mice were first trained to maintain balance for 2 minutes at a constant speed of 4 rpm. This training trial was followed by four test trials, during which the rod accelerated from 4 to 40 rpm in 5 minutes. Consecutive trials were separated by a 10 minute intertrial interval. Latency to falling off the rod was recorded up to 5 minutes. In the swim test, mice were placed in a circular pool (diameter 150 cm; water temperature 26°C) for 30 seconds. The animals were tracked using Ethovision tracking equipment and software (Noldus Bv, Wageningen, The Netherlands) for assessment of swimming velocity. Gait analysis was performed while mice were ventrally video-tracked on a transparent treadmill belt (Digigait, MouseSpecifics Inc, MA, USA). Following brief habituation to the apparatus, mice were tested at a constant belt speed of 16 cm/s. Different parameters were extracted from these video data using Digigait analysis software: base-widths (distance between contralateral paws), stride lengths (distance between subsequent placements of the same paw) and maximal paw area (peak stance). As an indicator of emotional function, an exploration test was performed in a plexiglass arena (42 cm × 26 cm × 30 cm) which mice could freely explore for 5 minutes. Mice were video-tracked with ANY-maze™ Video Tracking System software (Stoelting Co., IL, USA). Total distance travelled was calculated as a measure of exploratory activity. Finally, mice completed different learning and memory tasks. Working memory was assessed in a Y-maze consisting of 3 arms (5 cm wide, 30 cm long and enclosed by 30 cm high wall made of grey plastic) [32]. Mice were placed in the center for 10 minutes exploration of all arms. Locomotion was observed by a webcam connected to a screen. Entries into all arms were noted and an alternation was counted if an animal entered three different arms consecutively. Percentage spontaneous alternation was calculated according to following formula: [(number of alternations)/(total number of arm entries – 2)]* 100. Contextual and cued fear conditioning was evaluated in a plexiglass test chamber (26 × 22 × 18 cm high), containing a grid floor to deliver an electric shock using a constant current shocker (MED Associates Inc., St. Albans, Vermont, USA). The test set-up was placed inside a sound attenuated chamber. On the first day of the experiment, animals were placed in the testing chamber and allowed to acclimate for 5 minutes. On the second day, animals were again placed in the testing chamber and after 2 minutes of exploration (baseline score), a buzzer was sounded for 30 seconds. This auditory stimulus, the conditional stimulus, was followed by a 2 second foot shock (0.3 mA), the unconditional stimulus. After the shock, mice were allowed to explore once more for 1 minute before they received a second conditional–unconditional stimulus pairing. Finally, they were allowed to explore for another minute. Twenty-four hours later, on the third and last day, the animals were placed in the same context for 5 minutes exploration (contextual fear assessment). After 90 minutes, the mouse was again placed in the test chamber. Environmental and contextual cues were changed: a white plate was placed on the grid, light was switched on and minute extract was used to alter the smell. After 3 minutes of free exploration (pre-cue phase), the auditory stimulus was delivered for 3 minutes (cued fear assessment). Freezing behavior was recorded every 10 seconds during each trial block using the standard interval sampling procedure.

### Antibodies

Goat-Alexa Fluor® 488 and 594 conjugated anti-mouse or anti-rabbit secondary antibodies were used (Molecular Probes, Eugene, USA). Primary antibodies included rat-anti-CD68 (Serotec, Edinburgh, UK), rabbit-anti-actin, mouse-anti-GFAP, mouse-anti-MAP-2 (Sigma Aldrich, Steinheim, Germany), mouse-anti-huntingtin (Millipore, Schwalbach, Germany), rabbit-anti-LC3 and rabbit-anti-p62 (Enzo Life Sciences, Lörrach, Germany), rat-anti-LAMP-2 (Abl93) and rat-anti-LAMP-1 (1D4B) (DSHB, Iowa City, US), rabbit-anti-LAMP-2A (Pineda, Berlin, Germany), rabbit-anti-cathepsin D (a kind gift from Prof. J. Aerts), mouse-anti-MEF2D (BD Biosciences (Heidelberg, Germany), rabbit-anti-GAPDH and rabbit-anti-α-synuclein (C-20) (Santa Cruz, Dallas, US), rabbit-anti-caspase-3, rabbit-anti-phospho-PRAS40 and rabbit-anti-PRAS40 (Cell Signalling, Frankfurt am Main, Germany) and rabbit-anti-NSE (Abcam, Cambridge, UK).

### SDS-PAGE, Immunoblotting, assays monitoring MA and CMA substrate degradation and enzyme activity

For starvation assays, cells were extensively washed and cultured for 3 or 24 hours in Earl’s Balanced Salt Solution (EBSS) or Dulbecco's Modified Eagle's Medium (DMEM) without serum, supplemented with antibiotics before harvesting. Enzyme activity assays were performed on lysates from 54-week-old mice (n = 3 wild-type and n = 3 LAMP-2-deficient) as previously described [33]. N2a cells and brain tissue were lysed in RIPA buffer and denatured in Laemmli. For immunoblotting, protein samples were loaded on an SDS gel and blotted on a nitrocellulose or PVDF membrane (Roth, Karlsruhe, Germany). Membranes were probed with the relevant primary and secondary antibodies. Peroxidase-conjugated secondary antibodies were detected by chemiluminescence (SuperSignalWest, Pierce, Pittsburgh, USA). Densitometric analyses were performed with Image J software (http://imagej.nih.gov/ij/). Samples were normalized to the loading control and are presented as protein levels relative to wild-type or untreated, non-transfected samples where appropriate.

### Preparation and maintenance of primary neuronal and astroglial culture

Primary neurons were prepared as described previously [34] from the cortices of gestational day 15 embryos. After 3 days, cultured cells were treated with 10 μM cytosine arabinoside. Primary glial cultures were prepared as above but cultured in minimum essential medium with 10% horse serum and penicillin/streptomycin. Cells were fixed in 4% paraformaldehyde diluted in PBS after 14 days *in vitro*.

### Relative quantitative RT-PCR

Messenger RNA was isolated from whole brain using the NucleoSpin® RNAII kit (Macherey-Nagel, Düren, Germany) according to the manufacturer’s instructions. Complementary DNA was created using 2 μg total RNA and amplified using oligodT primers and the RevertAid cDNA synthesis Kit (Fermentas, St. Leon-Rot, Germany). Negative controls (RT-) were carried out in the absence of reverse transcriptase to check for genomic contamination. Quantitative RT-PCR, using actin as the house-keeping gene, was carried out using the Universal® probe library and Lightcycler 480 II from Roche (Mannheim, Germany) according to the manufacturer´s instructions. Relative expression was calculated using an efficiency-corrected comparative quantitation method [35]. To determine efficiency of each primer set, a standard curve was plotted using 10-fold serial dilutions of a mix of applied cDNA, followed by a logarithmic conversion and a linear response generated.

### Statistical analysis

All values are expressed as the mean ± standard error of the mean. Differences among mean values were analyzed via a two-tailed, unpaired Student *t-*test using Microsoft Excel software or one-way ANOVA followed by Tukey post-Hoc test, using GraphPad Prism 5, where multiple samples were compared. Behavioral data were analyzed with SigmaStat software using Student *t*-test Mann–Whitney Rank Sum Test or Repeated-Measures ANOVA where appropriate. The null hypothesis was rejected at p < 0.05.

## Results

### Loss of LAMP-2 expression causes neuropathological changes in mice

Similarities in the pathological presentation of Danon disease in cardiac and skeletal muscle of humans and LAMP-2-deficient (LAMP-2^-/y^) mice [4,28] that were of a mixed genetic background (SVJ-129/C57BL6-J (Harlan)) highlight the validity of this mouse as a model for the human disease. The neuropathological characterization of LAMP-2-deficient mice was carried out in animals backcrossed into C57/BL6-N (Charles River). We recently reported that backcrossing of LIMP-2 (Lysosomal Integral Membrane Protein type-2) knockout mice, that were maintained within the same mixed genetic background as the LAMP-2-deficient mice, into C57/BL6-N led to a deterioration of their phenotype including accumulation of α-syn, severe CNS impairments and premature death [36]. In contrast, LAMP-2-deficient mice of the mixed and C57/BL6-N background were macroscopically indistinguishable from each other.

**Figure 1:**
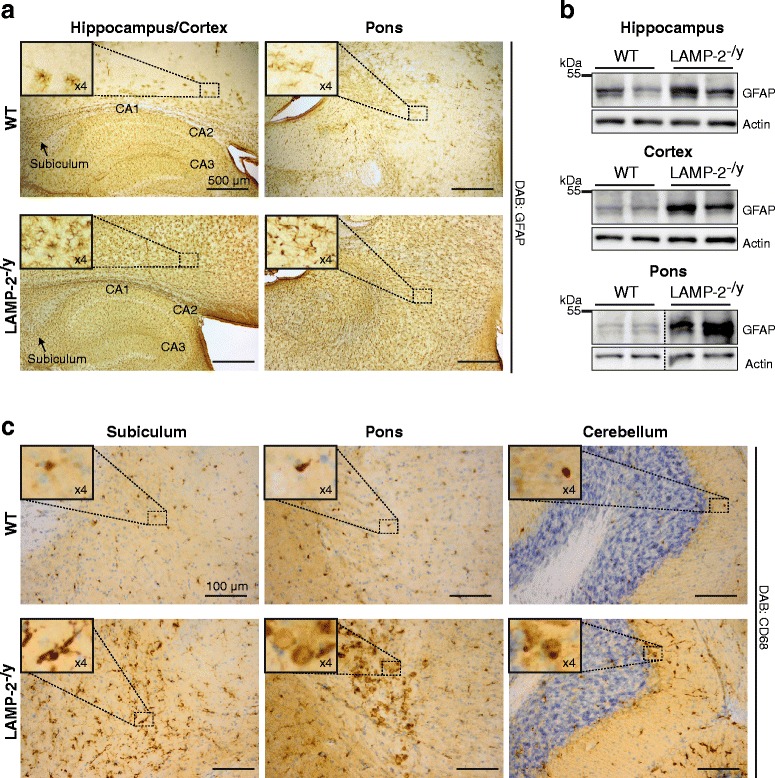
**Neuropathological changes in the absence of LAMP-2 expression.** Representative histological brain sections from LAMP-2-deficient (LAMP-2^-/y^) mice and their wild-type (WT) littermates (zoomed images shown in insets). **(a)** Astrogliosis in LAMP-2^-/y^ brain visualized with the aid of GFAP immunological staining. **(b)** Immunoblotting of brain lysates showing GFAP expression levels in WT and in LAMP-2^-/y^ brain tissue. **(c)** Microgliosis observed in LAMP-2^-/y^ animals via CD68 immunological staining (sections were costained with Nissl).

**Figure 2:**
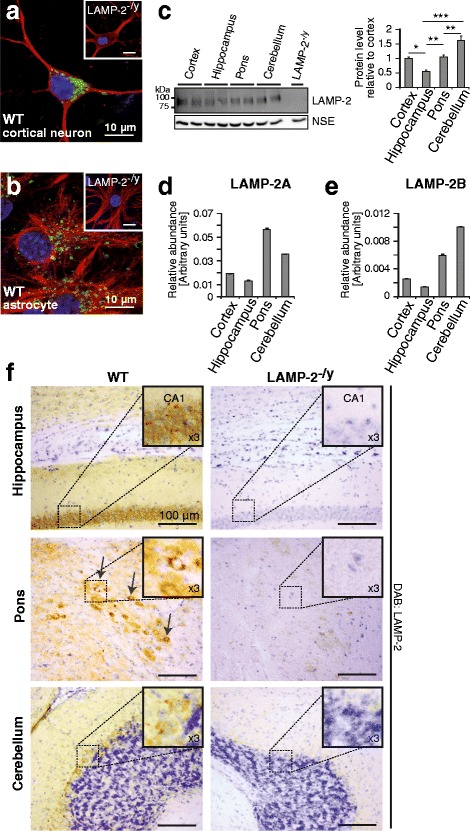
**LAMP-2 expression in brain.** LAMP-2 (green) vesicular staining detected in **(a)** MAP-2 (red) positive cultured primary neurons and **(b)** GFAP (red) positive primary astrocytes (LAMP-2-deficient (LAMP-2^-/y^) cultures were used to control for antibody specificity). **(c)** Immunoblot and respective densitometric analysis of LAMP-2 (n = 5) showing its expression throughout murine brain (cortex from a LAMP-2^-/y^ mouse was used to show specificity of the LAMP-2 antibody; actin was used as a loading control; *p < 0.05, **p < 0.01, ***p < 0.001). Quantitative RT-PCR of isoforms **(d)** LAMP-2A and **(e)** LAMP-2B in respective brain regions. **(f)** Representative histological brain sections stained for LAMP-2 using the DAB method and counterstained with Nissl. No signal was detected in LAMP-2^-/y^ brains. Enriched expression was observed in the pyramidal layer of the hippocampus, within the Purkinje cell layer of the cerebellum and within a region of the pons. Insets show zoomed images of regions outlined.

Histological analysis of the CNS of 12-month-old LAMP-2-deficient mice revealed wide-spread astrogliosis within all brain regions, including the hippocampus, as illustrated by immunohistochemistry (Figure 1a) and immunoblotting (Figure 1b) with an antibody directed against the Glial Fibrillary Acidic Protein (GFAP). Mild microgliosis (Figure 1c and Additional file 1a), as depicted by immunohistochemical staining using an antibody specific for the microglia resident protein macrosialin/CD68, was observed throughout LAMP-2-deficient brains. In the subiculum of the hippocampus and pons, microgliosis was most prominent. Here, microglia assumed a morphology indicative of their activation (Figure 1c, left and middle panels). Toluidine blue stained sections of LAMP-2-deficient brains, revealed darkly stained degenerating neurons within the hippocampus, particularly the subiculum and the CA3 region (Additional file 1b). However, no indication of apoptosis was evident, as highlighted by Terminal deoxynucleotidyl transferase dUTP Nick End Labeling (TUNEL) (Additional file 1c) and lack of caspase-3 cleavage (Additional file 1d). Using an antibody (Abl93, Development Studies Hybridoma Bank) that recognizes all LAMP-2 isoforms (pan LAMP-2), LAMP-2 was shown to be expressed in both primary cultured neurons (Figure 2a) and astrocytes (Figure 2b). Immunoblot analysis of different brain regions, obtained from wild-type mice, revealed ubiquitous expression of LAMP-2 within the CNS. Quantification of LAMP-2 immunoblot levels relative to cortex revealed the highest expression of LAMP-2 in the cerebellum and the lowest in the hippocampus (Figure 2c). Expression of both LAMP-2A and LAMP-2B isoforms in the hippocampus was also evident by quantitative (q) RT-PCR (Figure 2d/e). LAMP-2A appeared to be the most abundant isoform expressed in brain (Figure 2d) whereas transcripts of LAMP-2C, an isoform implicated in the uptake of RNA and DNA [37] was not detectable in brain using semi-quantitative PCR (data not shown). Immunohistochemistry of brain sections showed localized enriched expression of LAMP-2 in neurons especially in pyramidal neurons of the hippocampus (Figure 2f, upper panels and Additional file 2a, upper panels) and in the pons (Figure 2f, middle panels and Additional file 2a, lower panels). Despite its similar distribution, we did not observe a compensatory upregulation of LAMP-1 in LAMP-2-deficient brain as illustrated by LAMP-1 immunoblot (Additional file 2b) and immunohistological (Additional file 2c) analysis. From our data we conclude that LAMP-2 is an abundant protein of neuronal tissue and that loss of its expression causes neuroinflammation in mice.

### Behavioral anomalies as a result of LAMP-2 deficiency

**Figure 3:**
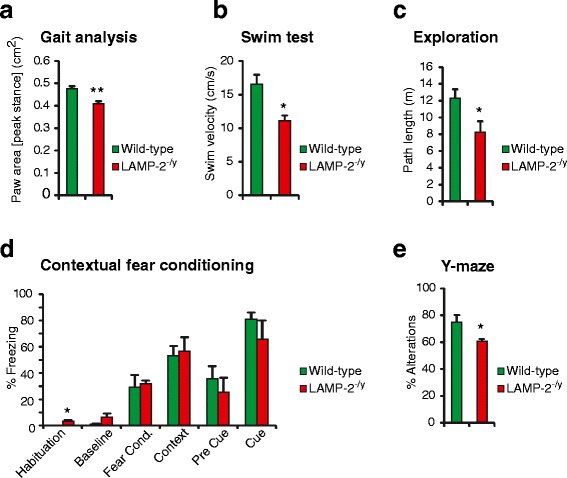
**Abnormal behavior as a result of LAMP-2 deficiency in mice. (a)** LAMP-2-deficient (LAMP-2^-/y^) mice show a significantly reduced paw area at the peak of stance in comparison with wild-type mice. **(b)** LAMP-2^-/y^ mice swim significantly slower than wild-type controls. **(c)** Path length during exploration of a novel environment is significantly reduced in LAMP-2^-/y^ mice. **(d)** Freezing percentage is significantly increased in LAMP-2^-/y^ mice during the habituation phase. There was no difference in fear conditioning and contextual or cued fear memory. **(e)** Y-maze working memory: LAMP-2^-/y^ mice make significantly less spontaneous alternations (*p < 0.05; **p < 0.01).

Danon disease patients present with neurological manifestations in the form of intellectual dysfunction [25-27]. LAMP-2-deficient mice were subjected to a battery of behavioral tests that included measures of motor performance (gait analysis, grip strength, activity, rotarod and swim test) and exploration as well as indices of learning and memory (Y-maze, contextual fear conditioning). Gross motor behavior appeared unaltered in LAMP-2-deficient mice, as there were no significant differences when compared to wild-type controls in grip strength, rotarod performance and home cage activity (Additional file 3a-c). Likewise, rudimentary gait parameters such as base-widths and stride lengths were similar between genotypes (data not shown). However, maximal paw area was reduced in LAMP-2-deficient mice (Figure 3a). Although this might indicate altered plantar placing in the context of a motor deficit, it is more likely a consequence of reduced body weight in these mice (data not shown). Impaired motor performance of LAMP-2-deficient mice was evident in the swim test exemplified by severely reduced swimming velocity (Figure 3b). This effect was not confirmed to be caused by muscular weakness as grip strength performance was normal. Reduced exploratory activity was observed in the exploration test (Figure 3c). Considering unaltered levels of home cage activity (Additional file 3c), this effect does not appear to reflect general hypolocomotion of LAMP-2-deficient mice. Rather, it could indicate blunted affective response to the novel environment. LAMP-2-deficient mice show an increased freezing percentage during the habituation phase of the contextual fear conditioning experiment, which may be the consequence of reduced exploratory movement. However, fear conditioning as well as contextual and cued fear memory was intact in LAMP-2-deficient mice (Figure 3d). In the Y-maze task, LAMP-2-deficient mice made relatively less alternations (Figure 3e) in comparison with wild-type control mice, indicating impaired working memory. In conclusion, LAMP-2-deficient mice show particular motor impairments, reduced exploratory activity and signs of impaired memory, consistent with hippocampal dysfunction.

### Lysosomal dysfunction in hippocampal neurons

**Figure 4:**
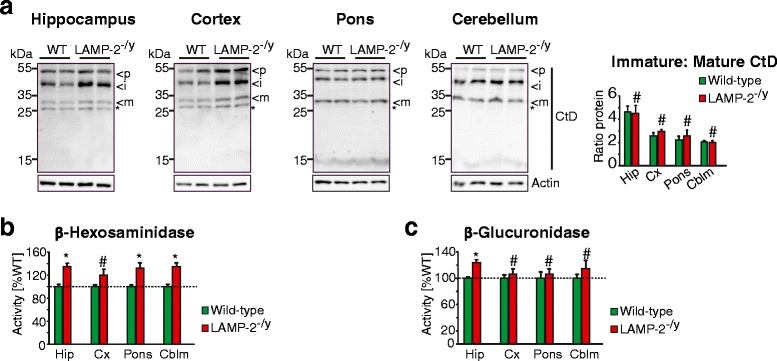
**Abnormal lysosomal activity in the absence of LAMP-2 expression. (a)** Immunoblots and respective densitometric quantification of cathepsin D (CtD) in wild-type (WT) or LAMP-2-deficient (LAMP-2^-/y^) brain lysates (data are shown as the mean; protein levels were normalized to actin, asterisk highlights a non-specific band recognized by the used cathepsin D antibody and p (premature), i (intermediate) and m (mature) denote the different forms of cathepsin D). **(b/c)** Regional specific activity of **(b)** β-hexosaminidase and **(c)** β-glucuronidase in wild-type and LAMP-2^-/y^ brain lysates (Hip, hippocampus; Cx, cortex; Cblm, cerebellum; ^#^p > 0.05, *p < 0.05).

The presence of neuroinflammation and behavioral anomalies in mice lacking LAMP-2 expression highlights the importance of this lysosomal protein in brain. To assess whether loss of LAMP-2 leads to lysosomal dysfunction that correlates with the observed neuropathological abnormalities, we determined enzyme activities of the lysosomal hydrolases β-hexosaminidase and β-glucuronidase as well as the maturation of cathepsin D as a means of evaluating lysosomal function. Whereas a significant increase of all forms of cathepsin D (pro (p), immature (i) and mature (m)) was observed in the hippocampus and in the cortex (Figure 4a, Additional file 4), no marked change in the maturation of the enzyme was detected (Figure 4a). Additionally, the hippocampus of LAMP-2 knockout mice displayed a significant increase in β-hexosaminidase (Figure 4b) and β-glucuronidase (Figure 4c) activity whereas in the pons and cerebellum only β-hexosaminidase activity was elevated. In summary, among all other brain regions investigated, the hippocampus of LAMP-2-deficient mice showed the most consistent signs of alterations in lysosomal enzyme activity.

### Lipid storage and autophagic vacuoles in LAMP-2-deficient hippocampal neurons

**Figure 5:**
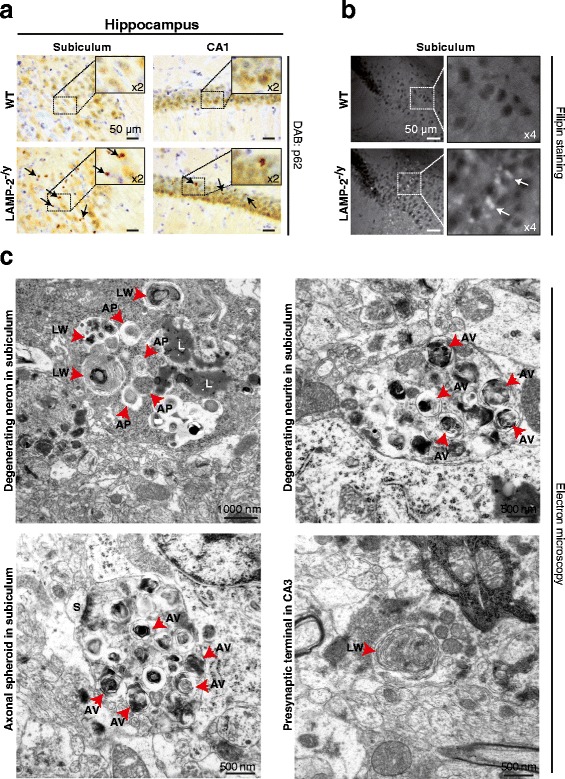
**Accumulation of p62 and filipin positive autophagic vesicles correlates with axonal and synaptic pathology in mice lacking LAMP-2.** Representative histological brain sections of wild-type (WT) and LAMP-2-deficient (LAMP-2^-/y^) mice stained for **(a)** p62 using DAB (sections were costained with Nissl; arrows point to p62-positive punctae; zoomed image shown to right of main images). **(b)** Filipin stained sections showing cholesterol storage within the subiculum of LAMP-2^-/y^ mice (insets show zoomed images of outlined area; arrows highlight storage). **(c)** High power electron micrographs from the subiculum and hippocampal CA3 region from LAMP-2^-/y^ mice. Neurites and presynaptic terminals showed numerous autophagosomes with storage material of different nature (arrows point to lipofuscin (L), autophagosomes (AP), autophagic vacuoles (AV) and lipid whorls (LW), S denotes a synaptic spine).

Degradation of long-lived proteins which has been shown to be impaired in LAMP-2-deficient hepatocytes [4] mainly occurs by autophagy [38]. The detection of increased levels of proteins that are pivotal for the autophagic flow and are degraded in the lysosome, such as the adaptor protein sequestosome 1 (SQSTM1/p62) [39,40], is widely used as an indication of impaired autophagy. Immunohistochemical analysis of LAMP-2-deficient brain revealed a distinct accumulation of p62-positive aggregates specifically within the subiculum and CA1 region of the hippocampus and the pons (Figure 5a).

Periodic-Acid-Schiff (PAS) staining identified carbohydrate-conjugates within the brain of one Danon disease patient [29]. In neuronal tissues of LAMP-2-deficient mice, no apparent PAS-positive storage was evident (Additional file 5a). Filipin staining displayed storage of free cholesterol exclusively in the subiculum of LAMP-2-deficient mice (Figure 5b). High power electron micrographs revealed the presence of Lipid Whorls (LW) that are implicated in lysosomal storage disease and cholesterol accumulation [41] in degenerating neurons within the CA3 and subiculum of LAMP-2-deficient mice (Figure 5c, upper left and lower right panels). Additionally, Lipofuscin (L) and Autophagic Vacuoles (AVs) containing amorphous or multilamellar material were evident within dystrophic neurites and axonal spheroids in the hippocampus (Figure 5c) which were not present in control mice (Additional file 5b). Storage of AVs within axons led to disruption of the presynaptic terminal highlighted by complete absence of synaptic vesicles (Figure 5c, bottom left panel). Storage was observed both within the soma and neurites of cells. Our histological data indicate that loss of LAMP-2 expression causes lysosomal/autophagic disturbances particularly in the hippocampus of mice. However, in hippocampal extracts that were prepared from LAMP-2 knockout mice we found no significantly increased levels of LC3-II or p62 (Additional file 5c). The ratio of phosphorylated proline-rich Akt substrate 40 (PRAS40) which is known to negatively regulate autophagy [42,43] was also not changed in LAMP-2-deficient samples (Additional file 5c, middle panel) indicating no overall increase in basal MA when LAMP-2 is absent. Proteasomal activity was also unaltered since levels of poly-ubiquitinated proteins were unchanged in LAMP-2 knockout brain samples when compared to wild-type controls (data not shown).

### Loss of LAMP-2 does not influence the steady-state levels of CMA substrates and is dispensable for starvation induced clearance of these proteins

**Figure 6:**
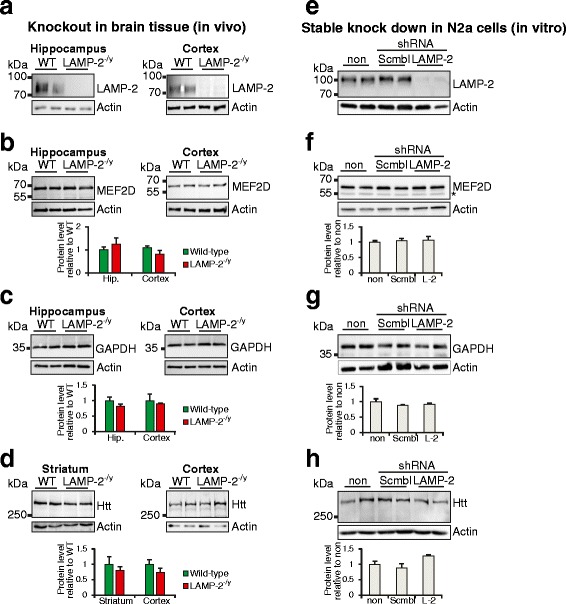
**Analysis of steady-state levels of CMA substrates in brain. (a-d)** Immunoblots and respective densitometry of lysed hippocampi/cortices from wild-type (WT) and LAMP-2-deficient (LAMP-2^-/y^) mice showing protein levels of LAMP-2 **(a)**, MEF2D **(b)**, GAPDH **(c)** and huntingtin (Htt) **(d)**. **(e-h)** Immunoblots and respective densitometry of lysates from N2a cells either non-transfected (non) or stably transfected with scramble shRNA (scmbl) or shRNA targeting LAMP-2 mRNA showing steady-state levels of LAMP-2 **(e)**, MEF2D **(f)**, GAPDH **(g)** or Htt **(h)** (actin was used to control loading; *denotes a non-specific band for MEF2D). LAMP-2 levels were not affected by scramble (scmbl) shRNA.

LAMP-2A is reported to be the rate-limiting factor in CMA [14,15]. Such proteins that are degraded via CMA include those associated with neurodegenerative diseases such as MEF2D, Htt as well as the glycolytic enzyme GAPDH [15]. Thus, we assessed a potential accumulation of these substrates, due to the lack of the CMA receptor LAMP-2A, through analysis of brain lysates from knockout animals as well as *in vitro* after stable knockdown of LAMP-2 in murine neuroblastoma cells (N2a). Absence of LAMP-2 expression in cortex and hippocampus (Figure 6a) did not affect the steady-state levels of MEF2D (Figure 6b), GAPDH (Figure 6c) or Htt (Figure 6d, Additional file 6a). Expression levels of Htt were also found to be unchanged in striatum (Figure 6d) which is the primary site of neuronal damage in Huntington`s disease. Similarly, stable knockdown of LAMP-2 in N2a cells using shRNA directed against LAMP-2 mRNA significantly reduced protein levels of LAMP-2 (Figure 6e) but not LAMP-1 (Additional file 6b) and did not alter steady-state levels of MEF2D (Figure 6f), GAPDH (Figure 6g) or Htt (Figure 6h). Furthermore, an accumulation of MEF2D within the cytosol, which has been reported after blockage of CMA by LAMP-2 silencing [44], was not revealed neither in LAMP-2-deficient primary neurons (Additional file 6c) nor in brain sections (Additional file 6d). The typical nuclear localization of this protein was observed independent of the expression of LAMP-2.

**Figure 7:**
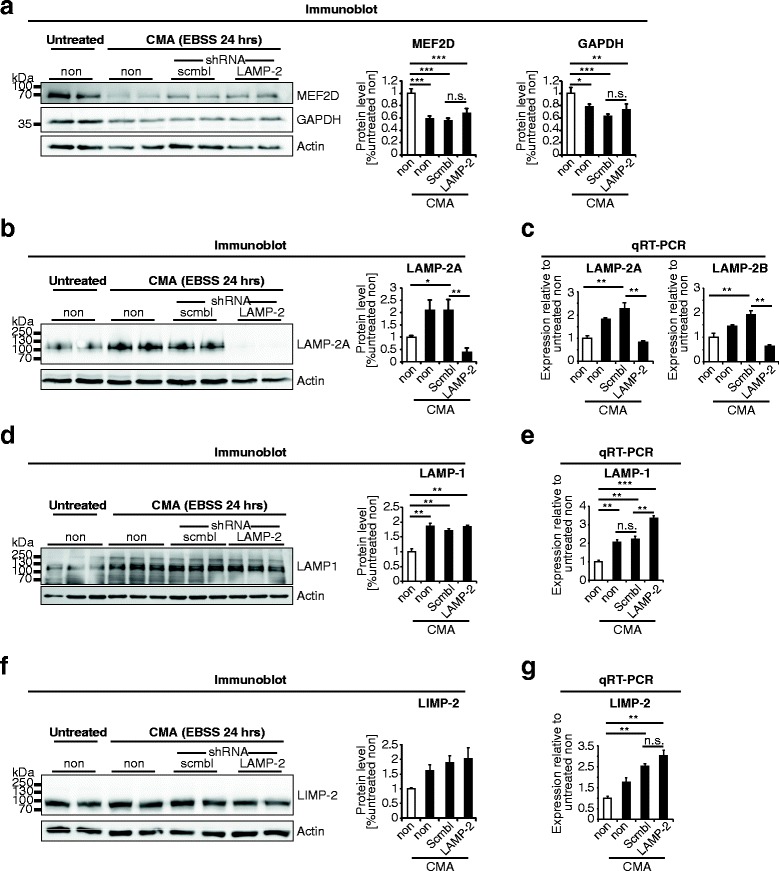
**Lysosomal protein content and CMA substrate steady-state levels after prolonged starvation.** Immunoblotting and quantitative RT-PCR of lysates from N2a cells either non-transfected (non) or stably transfected with scramble shRNA (scmbl) or shRNA targeting LAMP-2 mRNA. Cells were cultured in EBSS media for 24 hours to induce CMA. **(a)** Immunoblots and respective densitometric quantification showing protein levels of CMA substrates MEF2D and GAPDH **(b)** Immunoblots and respective densitometric quantification of LAMP-2A and **(c)** quantitative RT-PCR of LAMP-2A and LAMP-2B. **(d)** Immunoblots and respective densitometric quantification showing protein levels of LAMP-1. **(e)** Quantitative RT-PCR of LAMP-1. **(f)** Immunoblots and respective densitometric quantification showing protein levels of LIMP-2. **(g)** Quantitative RT-PCR of LIMP-2. (Samples were cultured in EBSS for 24 hours to induce CMA; actin was used to control loading; *p < 0.05, **p < 0.01, ***p < 0.001).

Prolonged starvation of cells for 24 hours in serum-free media is known to induce CMA [45]. In order to investigate LAMP-2-dependent clearance of substrates after extended starvation, N2a cells, stably transfected with either LAMP-2 shRNA or scramble (control) shRNA, were cultured in nutrient free (without serum and amino acids) Earl`s Balanced Salt Solution (EBSS) or in Dulbecco`s Modified Eagle Medium (DMEM) devoid of serum for 24 hours. Both starvation treatments did not affect the viability of cells as judged by visual inspection and immunoblotting of cleaved caspase-3 (data not shown). Since prolonged starvation under nutrient free conditions induces expression and nuclear translocation of the transcription factor EB (TFEB), that is known to modulate lysosomal biogenesis [46], we have assessed protein levels of LAMP-2A, LAMP-2B, LAMP-1 and LIMP-2 via immunoblotting and qRT-PCR analysis. In order to specifically detect LAMP-2A we have used a self-made antibody that only recognizes LAMP-2A as highlighted by immunoblots of HeLa cell extracts overexpressing the three different LAMP-2 isoforms (LAMP-2A, B and C) as well as LAMP-1. Furthermore, no signal was detectable in LAMP-2-deficient brains, confirming the specificity of this antibody (Additional file 7a). Serum removal for 24 hours decreased steady-state levels of MEF2D, independent of the expression of LAMP-2 (Additional file 7b). Prolonged starvation in complete nutrient-free medium also led to a pronounced decline of MEF2D levels as well as GAPDH (Figure 7a) independent of LAMP-2 expression (Figure 7b). Messenger RNA levels of GAPDH and MEF2D were equal in control and LAMP-2 shRNA starved samples (Additional file 7c).

Under these conditions we observed a significant increase in LAMP-2A expression in control cells as shown by immunoblotting (Figure 7b) and qRT-PCR (Figure 7c). Interestingly, an increase was also observed in LAMP-2B (Figure 7c) as well as two other lysosomal membrane proteins investigated namely LAMP-1 (Figure 7d/e) and LIMP-2 (Figure 7f/g).

**Figure 8:**
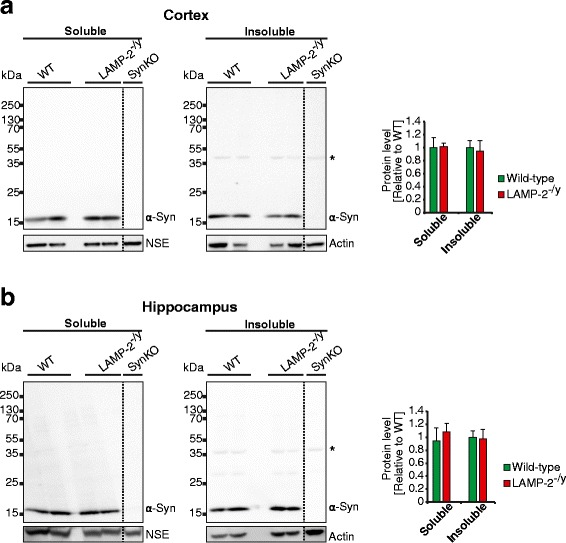
**α-synuclein is not accumulated in the absence of LAMP-2 expression. (a/b)** Immunoblots and respective densitometric quantification of soluble and insoluble fractions from cortices **(a)** and hippocampi **(b)** of LAMP-2-deficient (LAMP-2^-/y^) mice and their wild-type (WT) littermates. The asterisk highlights a non-specific band recognized by the α-syn antibody (C-20, Santa Cruz; NSE or actin were used as loading controls).

The knockdown of LAMP-2 did not alter basal (Additional file 7d) or induced MA (Additional file 7e) as indicated by unchanged levels of phosphorylated PRAS40, p62 and LC3-II in control and LAMP-2 shRNA treated N2a cells. Successful induction of MA was monitored by a decline in p62 levels and reduced phosphorylation of PRAS40 (Additional file 7e). Finally, despite the fact that down-regulation of LAMP-2 was reported to lead to an accumulation of another CMA substrate, α-syn in cultured neuronal cells [19,47], complete loss of LAMP-2 expression in murine brain did not cause a significant accumulation of either monomeric or high-molecular weight species of α-syn within the cortex or hippocampus (Figure 8a/b).

Though prolonged starvation significantly increased LAMP-2A levels in N2a cells, this is not unique to this isoform since a comparable upregulation was also evident for LAMP-2B, LAMP-1 and LIMP-2. Furthermore, absence of LAMP-2 did not affect CMA substrate levels in brain tissue and in neuroblastoma cells.

## Discussion

The current report highlights LAMP-2 as a ubiquitously expressed protein of the CNS. In addition to its established role as a receptor for CMA, the function of LAMP-2 in the brain is not yet well defined. Absence of LAMP-2 in brain, especially within the subiculum and CA3 region of the hippocampus, caused pronounced inflammation as well as perturbed lysosomal activity and autophagy indicated by intraneuronal lipid storage, accumulation of autophagic vacuoles and neuronal degeneration in a subpopulation of neurons. Hippocampal neurons are particularly vulnerable to lysosomal storage induced degeneration as highlighted in various mouse models with lysosomal dysfunction [48,49] and in Alzheimer and Parkinson`s disease as well as dementia with Lewy bodies. To some extent this vulnerability can be explained by regional differences in the expression profile of proteins that can induce neurotoxicity under certain pathophysiological conditions such as aggregation of α-syn and amyloid-β. Interestingly, hippocampal but not cortical motor neurons were described as vulnerable to ischemia/reactive oxygen species-induced neurotoxicity through calpain-mediated cleavage of carbonylated hsp70 [50] thereby diminishing its neuroprotective effect [51,52]. A reduction in LAMP-2 expression has been directly linked to low CMA activity as well as increased levels of oxidized proteins in aged livers [15,45] and breast cancer cells [53]. It is therefore tempting to speculate that loss of LAMP-2, especially within hippocampal neurons, leads to an accumulation of oxidized proteins that may contribute to the observed neuropathology. Further studies are necessary in order to elucidate the impact of oxidative stress as a potential cause of CMA blockage on the neuropathology of LAMP-2-deficient mice.

Our data suggest a distinct role of LAMP-2 within the hippocampus that might be causative for the observed intellectual dysfunction in Danon disease patients [25,26,54,55]. Besides the development of a mild astrogliosis, no other neuropathological changes within the hippocampus were described within the brain of one Danon disease patient [29]. Therefore, the hippocampus should be of special interest when examining brain autopsy material from Danon disease patients in the future. It should also be noted that according to our analyses, LAMP-2A is the most prominent isoform in brain whereas LAMP-2B was only present to a minor degree and LAMP-2C was undetectable under the experimental conditions used. This is in contrast to the described prominent expression of LAMP-2C in murine brain [37]. Therefore, further analysis is necessary in order to elucidate the expression and relative abundance of the different LAMP-2 isoforms in brain.

Due to the rarity of the disease and the limited access to autopsy brain material, LAMP-2-deficient mice provide an important tool in studying the molecular mechanisms underlying Danon disease neuropathology. We observed behavioral changes in these mice which relate to neuromuscular and intellectual dysfunction in patients. Likewise, the neuropathology of LAMP-2-deficient mice and Danon disease patients share common characteristics. Both show pronounced inflammation exemplified by an increased expression of GFAP in activated astrocytes despite absence of obvious signs of pronounced neurodegeneration [29]. Interestingly, GFAP was identified as a modulator of CMA that stabilizes the multimeric CMA translocation complex formed within the lysosomal membrane after CMA induction [56]. Absence of LAMP-2 may interfere with formation of such a complex ultimately leading to the activation of astrocytes.

In addition to inflammation, another prominent feature of the neuropathology described for one Danon disease patient is the presence of electron dense bodies and lipofuscin-rich deposits throughout the brain [29]. In mice, among all other brain regions investigated, the hippocampus displayed most prominent signs of storage especially in the subiculum in which neuronal accumulation of free cholesterol and lipofuscin was evident. Previously, we reported cholesterol storage in LAMP-2-deficient liver [10]. This phenotype was mirrored in murine embryonic fibroblasts lacking LAMP-2 and to a higher extent in cells in which both LAMP proteins were deleted [9,10]. Rescue experiments of the observed cholesterol storage by re-expressing both LAMPs into LAMP-1/LAMP-2-double-deficient fibroblasts suggest a distinct role of LAMP-2 but not LAMP-1 in cholesterol metabolism. However, this effect was not isoform specific since all three LAMP-2 variants were equally effective in abolishing the late endosomal/lysosomal cholesterol accumulation [10]. Until now, the precise role of LAMP-2 within this process remains unknown and further analysis is necessary in order to clarify how its expression influences lipid and cholesterol metabolism, especially within neuronal cells.

The pathological hallmark of Danon disease, which was classified as a glycogen storage disorder [25,57], is the presence of accumulated glycogen and autophagic vacuoles within liver and muscle of patients and the mouse model [4,26,58]. Therefore, LAMP-2 was suggested to play an important role in the clearance of glycogen and maturation of autophagic vacuoles [5,59]. Interestingly, the glycogen storage disorders Lafora and Pompe disease, caused by mutations within enzymes directly or indirectly involved in glycogen metabolism [57], result in pronounced impairment of autophagy in tissues that accumulated glycogen, including the CNS [60-63]. Therefore, absence of accumulated glycogen within brain samples of LAMP-2-deficient mice may explain the lack of gross abnormalities in neuronal autophagy and suggest a distinct function of LAMP-2 within the hippocampus for autophagic clearance of neuronal proteins.

The most well defined role for LAMP-2A is its function as a receptor for CMA, mediating the lysosomal uptake of selective substrates such as MEF2D [44], Htt [22,64,65], α-syn [47] and GAPDH [15]. In our hands, despite apparent unaltered levels of MA and no change in proteasomal activity, loss of LAMP-2 expression did not affect steady-state levels of these proteins in brain lysates or N2a cells under normal conditions. A compensatory upregulation of MA and proteasomal activity that has been found upon blockage of CMA by silencing LAMP-2 expression [66] is therefore unlikely to account for the unchanged levels of CMA substrates in LAMP-2-deficient brain. Of note, we also did not observe cytosolic accumulation of MEF2D which has been reported as consequence of CMA blockage by knock-down of LAMP-2 expression [44]. In our hands, neither complete absence nor down-regulation of LAMP-2 affected the typical nuclear localization of this protein further suggesting that LAMP-2 is dispensable for clearance of CMA substrates under the investigated conditions. However, lysosomal uptake experiments of selected CMA substrates would help to clarify the impact of LAMP-2 deficiency within this pathway under normal and stress induced conditions.

With regard to the unchanged levels of α-syn in LAMP-2 knockout brains it is interesting to note that elevated expression of α-syn in the C57/BL6-N genetic background led to a deterioration of the phenotype of LIMP-2-deficient mice [36]. In contrast, absence of LAMP-2 within the same genetic background did not affect α-syn levels suggesting that LAMP-2 is dispensable for its clearance. This correlates with previous studies where double deficiency of LAMP-1 and 2 in mouse embryonic fibroblasts did not affect CMA-mediated proteolysis, suggesting existence of alternative receptors or mechanisms for CMA besides LAMP-2A [9,67].

CMA can be induced indirectly by various stimuli such as prolonged starvation or directly by overexpression of its receptor LAMP-2A [68]. Htt, MEF2D, GAPDH and α-syn levels were shown by others to be directly linked to expression of LAMP-2A [19,20,44,47]. However, in N2a cells, similar to the situation in LAMP-2-deficient brains, a reduction in LAMP-2 expression did not affect steady-state levels of CMA substrates even after prolonged incubation in nutrient or serum-free medium. Furthermore, in control cells, prolonged starvation did not specifically elevate LAMP-2A levels but led to a general increase in lysosomal membrane protein expression namely LAMP-2B, LAMP-1 and LIMP-2. Interestingly, this is in agreement with reports where an increase in LAMP-2A but also LAMP-1 expression was reported after prolonged starvation [15]. Our results are in agreement with the described effect of prolonged starvation that stimulates TFEB induced lysosomal biogenesis and degradation by increasing the expression of lysosomal membrane proteins and hydrolases [46,69]. However, further analysis will aid in the interpretation of the role of TFEB in starvation-induced CMA and its effect on expression levels, specifically of LAMP-2A.

## Conclusion

In conclusion, our data suggest a novel role of LAMP-2 for lysosomal and autophagic clearance within hippocampal neurons and contribute to the understanding of neuropathology in Danon disease.

## Additional files

Additional file 1:
**Neuropathology of LAMP-2-deficient mice.** Representative histological brain sections from LAMP-2-deficient (LAMP-2^-/y^) mice and their wild-type (WT) littermates. (a) Microgliosis observed in LAMP-2^-/y^ animals via CD68 immunological staining (insets depict zoomed images of outlined area, sections were costained with Nissl). Representative histological sections stained with (b) toluidine blue (arrows highlight shrunken cells) and (c) TUNEL (sections were costained with the nuclear stain haematoxylin). (d) Immunoblot of caspase-3 from hippocampal lysates (FL, full length, p17 and p20 refer to caspase-3 cleavage products; NSE was used to control loading). Additional file 2:
**No change in LAMP-1 expression in absence of LAMP-2 within brain.** (a) Representative histological brain sections stained for LAMP-2 using the DAB method and counterstained with Nissl. No signal was detected in LAMP-2-deficient (LAMP-2^-/y^) brains. Enriched expression was observed in an area of the pons. (b) Immunoblots of lysates from wildtype (WT) and LAMP-2^-/y^ brains probed for LAMP-1 and respective densitometric quantification (actin was used to control loading). (c) Representative histological brain sections stained for LAMP-1 using the DAB method and counterstained with Nissl. Enriched expression was observed in the pyramidal layer of the hippocampus, within the Purkinje cell layer of the cerebellum and within a region of the pons. Zoomed imaged of regions outlined are shown underneath pictures of the hippocampus/pons (#p>0.05). Additional file 3:
**Behavioral analyses of mice at 54 weeks.** (a) LAMP-2-deficient (LAMP-2^-/y^) mice showed similar grip strength when compared to wild-type controls. (b) No significant difference in performance using a rotarod was observed between wild-type and LAMP-2^-/y^ mice. (c) No differences in cage activity were observed between wild-type and LAMP-2^-/y^ mice. Additional file 4:
**Cathepsin D levels in brain.** Densitometric analysis of cathepsin D (CtD) levels corresponding to representative blots in Figure 4 (#p>0.05, *p<0.05, **p<0.01). Additional file 5:
**Absence of carbohydrate storage or perturbed autophagy in LAMP-2-deficient brain.** (a) Representative histological sections from LAMP-2-deficient (LAMP-2^-/y^) mice and their wild-type (WT) littermates stained with Periodic-Acid-Schiff (PAS). Sections were costained with the nuclear stain haematoxylin. (b) Electron micrograph showing normal morphology within the subiculum of a WT animal. (c) Immunoblots and respective densitometric quantification of hippocampal lysates. Additional file 6:
**Lack of LAMP-2 expression does not affect LAMP-1 levels and has no effect on huntingtin or MEF2D.** (a) Immunoblot of hippocampal lysates from LAMP-2-deficient (LAMP-2^-/y^) mice and their wild-type (WT) littermates. (b) Immunoblots of lysates from N2a cells either non-transfected (non) or stably transfected with scramble shRNA (Scmbl) or shRNA targeting LAMP-2 mRNA showing protein levels of LAMP-1. The specificity of the applied shRNA LAMP-2 probe is highlighted by unchanged levels of LAMP-1 protein (actin was used to control loading). (c) Immunofluorescence of MEF2D (red) and LAMP-2 (green) in primary neurons. (d) Immunofluorescence of MEF2D (red) in hippocampal slices (Dapi (4',6-diamidino-2-phenylindole) was used as a nuclear stain, insets shown zoomed images of area outlined). Additional file 7:
**Stable knockdown of LAMP-2 has no effect on expression of MEF2D or GAPDH in N2a cells.** (a) Immunoblot of lysates from brain lysates from wild-type (WT) and LAMP-2-deficient (LAMP-2^-/y^) mice as well as HeLa cells overexpressing eGFP, murine LAMP-1 (L1), murine LAMP-2A (L2A), murine LAMP-2B (L2B) or murine LAMP-2C (L2C). The LAMP-2A antibody used in this study is specific for the murine isoform A (actin was used to control loading and LAMP-1 to control for specificity). (b) Immunoblots and respective quanticiation from N2a cells either non-transfected (non) or stably transfected with scramble shRNA (scmbl) or shRNA targeting LAMP-2 mRNA. MEF2D levels decrease after starvation in serum-free DMEM. (c) Quantitative RT-PCR of samples from N2a cells either non-transfected (non) or stably transfected with scmbl shRNA or shRNA targeting LAMP-2 mRNA. MEF2D expression increases after starvation of the cells. However, no significant differences were observed between scmbl and LAMP-2 shRNA treated samples. GAPDH expression remained constant in all samples (#p>0.05, ***p<0.001). (d) Immunoblot and respective quantification from N2a cells showing lack of changes in macroautophagy upon knockdown of LAMP-2 (p-PRAS40 refers to phosphorylated-PRAS40; actin was used to control loading). (e) Immunoblots and respective densitometric quantification of lysates from N2a cells either untreated or starved for 3 hours to induce macroautophagy.

## Abbreviations

CMA: Chaperone-mediated autophagy
Htt: Huntingtin
α-syn: α-synuclein
MEF2D: Myocyte-specific enhancer factor-2D
GAPDH: Glyceraldehyde-3-phosphate dehydrogenase
LAMP-2: Lysosomal associated membrane protein type-2
MA: Macroautophagy
