# Supplementary figures and images for: LAMP-2 deficiency leads to hippocampal dysfunction but normal clearance of neuronal substrates of chaperone-mediated autophagy in a mouse model for Danon disease
Source: Acta Neuropathol Commun. 2015 Jan 31;3:6. doi: 10.1186/s40478-014-0182-y (PMC4359523; doi:10.1186/s40478-014-0182-y)

**a**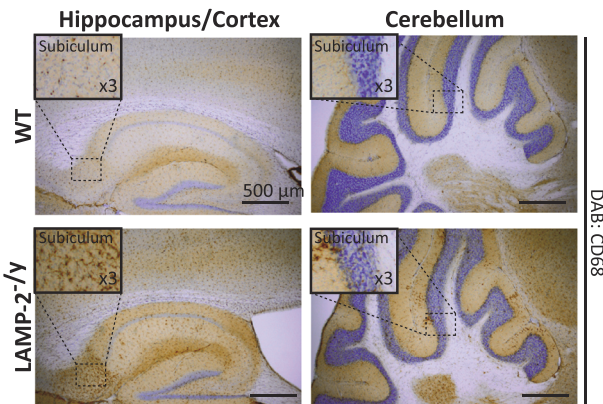**b**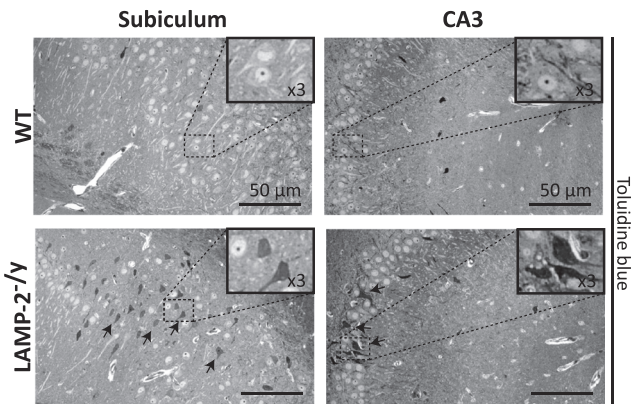**c**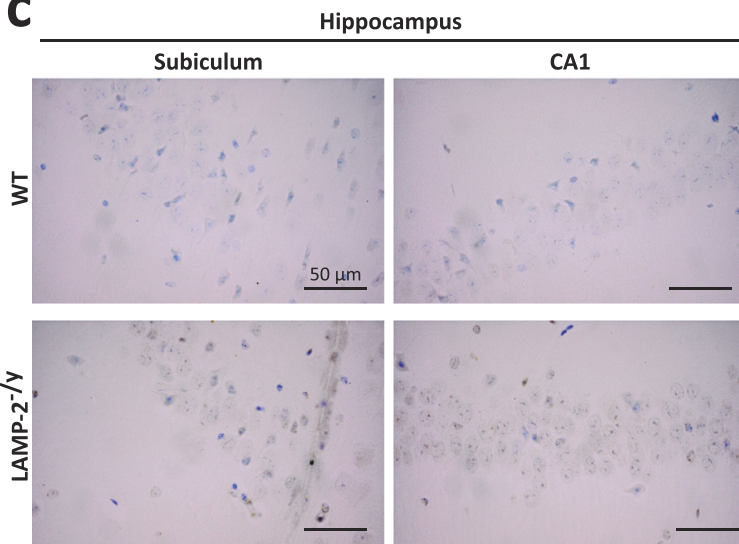**d**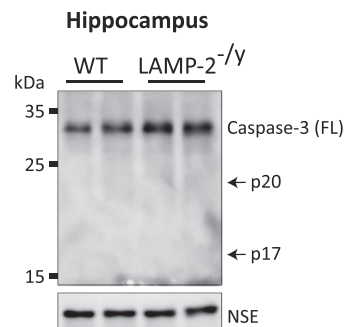

Supplement: Additional file 1: — Neuropathology of LAMP-2-deficient mice. Representative histological brain sections from LAMP-2-deficient (LAMP-2-/y) mice and their wild-type (WT) littermates. (a) Microgliosis observed in LAMP-2-/y animals via CD68 immunological staining (insets depict zoomed images of outlined area, sections were costained with Nissl). Representative histological sections stained with (b) toluidine blue (arrows highlight shrunken cells) and (c) TUNEL (sections were costained with the nuclear stain haematoxylin). (d) Immunoblot of caspase-3 from hippocampal lysates (FL, full length, p17 and p20 refer to caspase-3 cleavage products; NSE was used to control loading). [file 40478_2014_182_MOESM1_ESM.pdf]

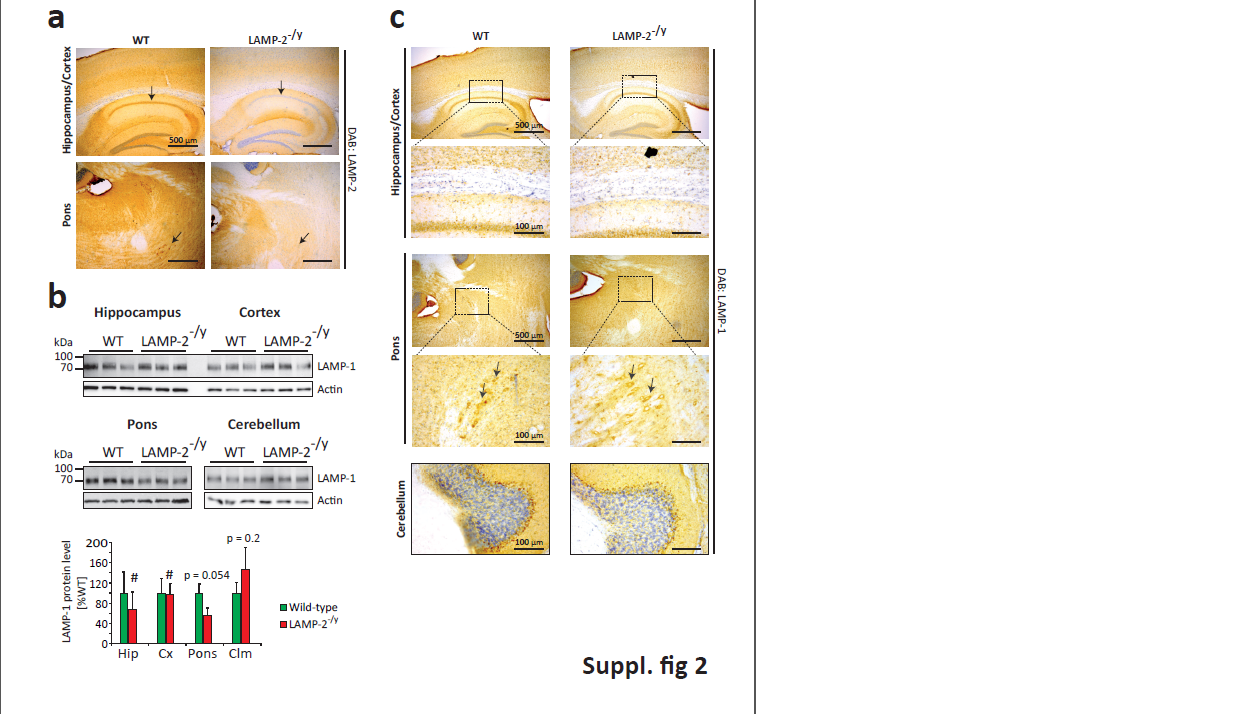

Supplement: Additional file 2: — No change in LAMP-1 expression in absence of LAMP-2 within brain. (a) Representative histological brain sections stained for LAMP-2 using the DAB method and counterstained with Nissl. No signal was detected in LAMP-2-deficient (LAMP-2-/y) brains. Enriched expression was observed in an area of the pons. (b) Immunoblots of lysates from wildtype (WT) and LAMP-2-/y brains probed for LAMP-1 and respective densitometric quantification (actin was used to control loading). (c) Representative histological brain sections stained for LAMP-1 using the DAB method and counterstained with Nissl. Enriched expression was observed in the pyramidal layer of the hippocampus, within the Purkinje cell layer of the cerebellum and within a region of the pons. Zoomed imaged of regions outlined are shown underneath pictures of the hippocampus/pons (#p>0.05). [file 40478_2014_182_MOESM2_ESM.png]

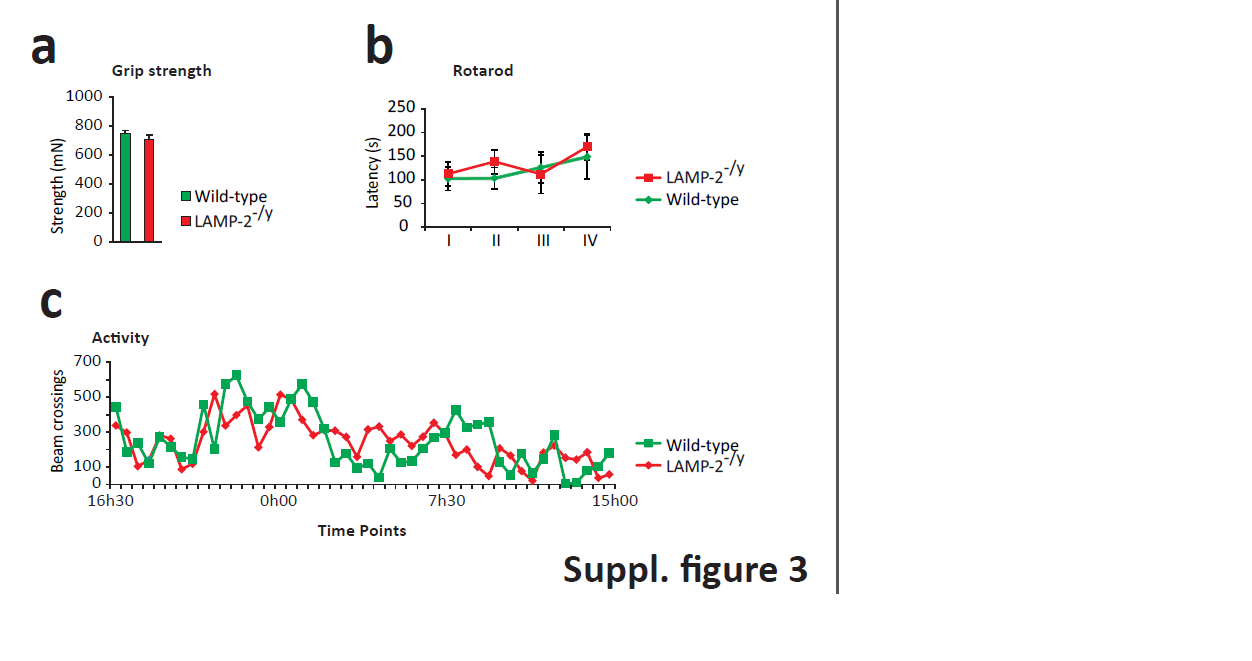

Supplement: Additional file 3: — Behavioral analyses of mice at 54 weeks. (a) LAMP-2-deficient (LAMP-2-/y) mice showed similar grip strength when compared to wild-type controls. (b) No significant difference in performance using a rotarod was observed between wild-type and LAMP-2-/y mice. (c) No differences in cage activity were observed between wild-type and LAMP-2-/y mice. [file 40478_2014_182_MOESM3_ESM.png]

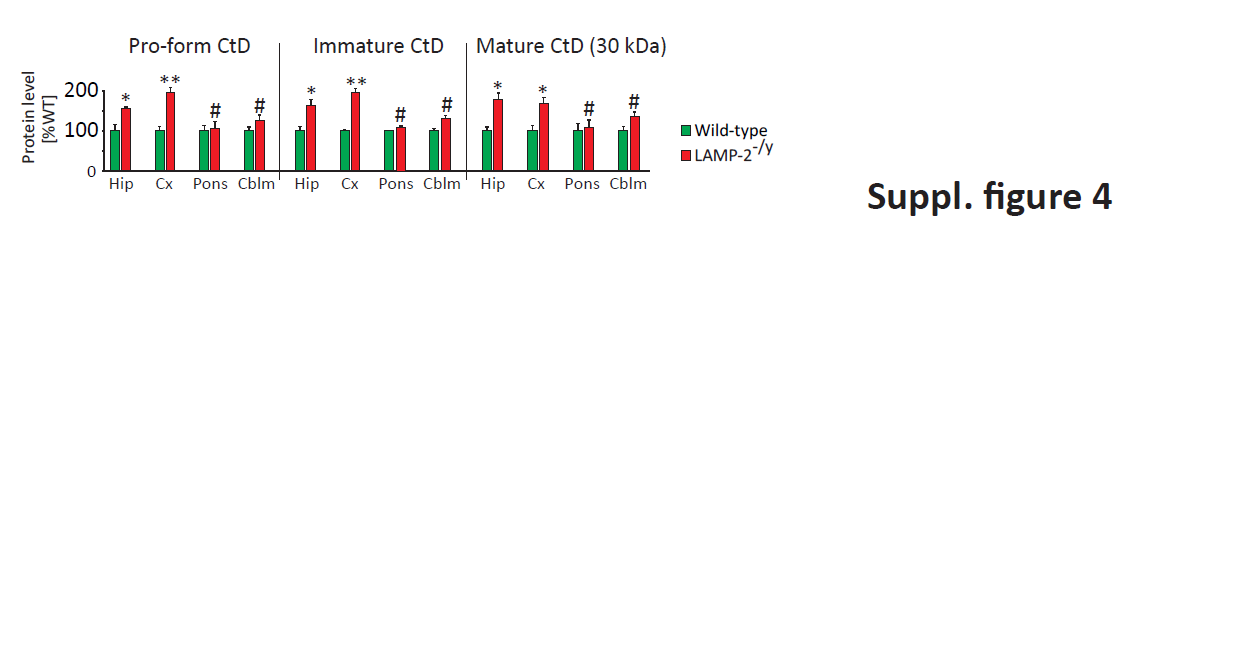

Supplement: Additional file 4: — Cathepsin D levels in brain. Densitometric analysis of cathepsin D (CtD) levels corresponding to representative blots in Figure 4 (#p>0.05, *p<0.05, **p<0.01). [file 40478_2014_182_MOESM4_ESM.png]

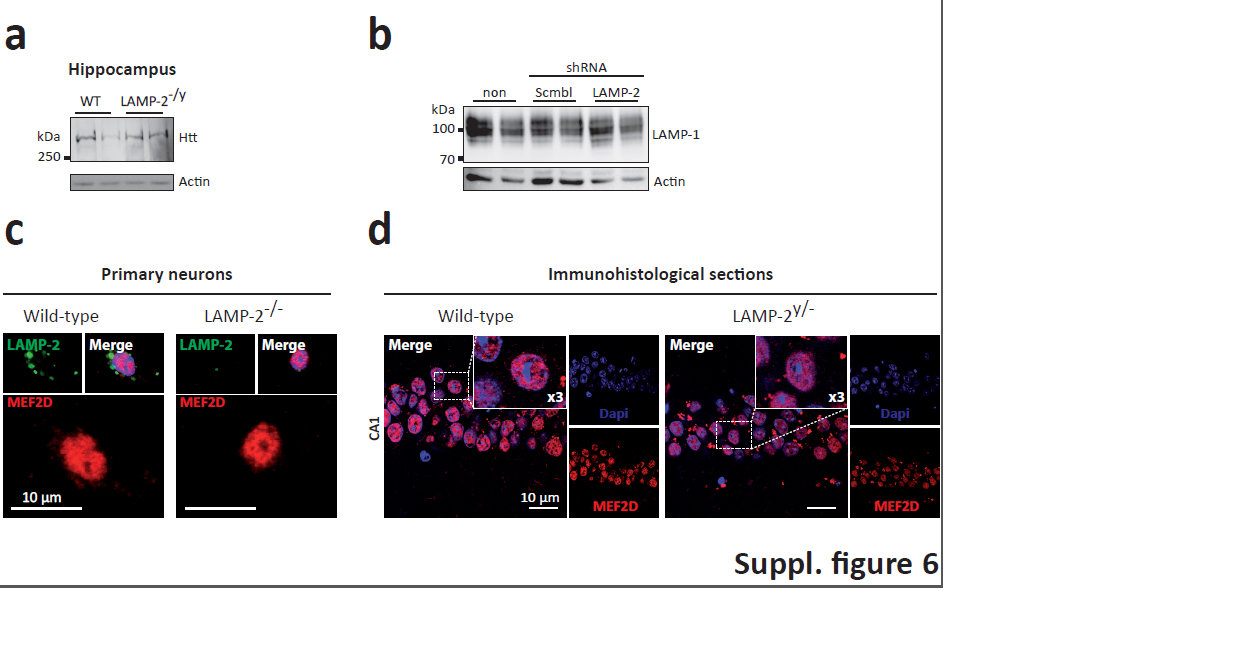

Supplement: Additional file 6: — Lack of LAMP-2 expression does not affect LAMP-1 levels and has no effect on huntingtin or MEF2D. (a) Immunoblot of hippocampal lysates from LAMP-2-deficient (LAMP-2-/y) mice and their wild-type (WT) littermates. (b) Immunoblots of lysates from N2a cells either non-transfected (non) or stably transfected with scramble shRNA (Scmbl) or shRNA targeting LAMP-2 mRNA showing protein levels of LAMP-1. The specificity of the applied shRNA LAMP-2 probe is highlighted by unchanged levels of LAMP-1 protein (actin was used to control loading). (c) Immunofluorescence of MEF2D (red) and LAMP-2 (green) in primary neurons. (d) Immunofluorescence of MEF2D (red) in hippocampal slices (Dapi (4',6-diamidino-2-phenylindole) was used as a nuclear stain, insets shown zoomed images of area outlined). [file 40478_2014_182_MOESM6_ESM.png]

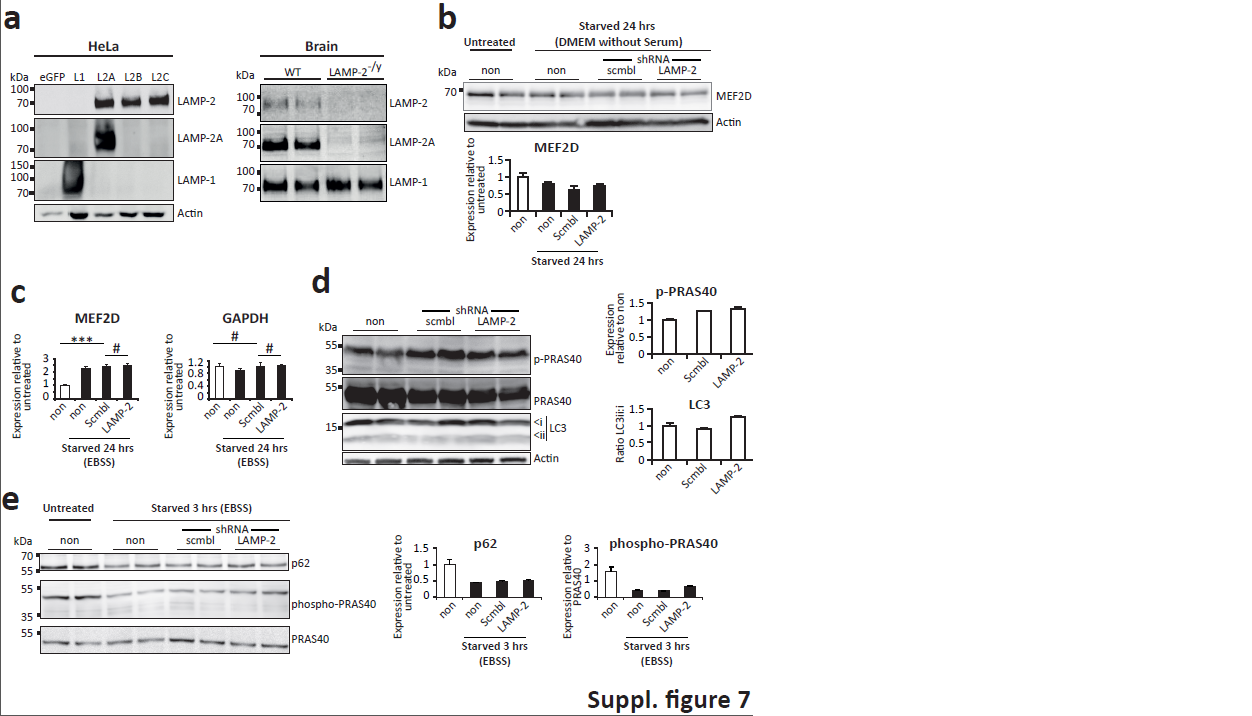

Supplement: Additional file 7: — Stable knockdown of LAMP-2 has no effect on expression of MEF2D or GAPDH in N2a cells. (a) Immunoblot of lysates from brain lysates from wild-type (WT) and LAMP-2-deficient (LAMP-2-/y) mice as well as HeLa cells overexpressing eGFP, murine LAMP-1 (L1), murine LAMP-2A (L2A), murine LAMP-2B (L2B) or murine LAMP-2C (L2C). The LAMP-2A antibody used in this study is specific for the murine isoform A (actin was used to control loading and LAMP-1 to control for specificity). (b) Immunoblots and respective quanticiation from N2a cells either non-transfected (non) or stably transfected with scramble shRNA (scmbl) or shRNA targeting LAMP-2 mRNA. MEF2D levels decrease after starvation in serum-free DMEM. (c) Quantitative RT-PCR of samples from N2a cells either non-transfected (non) or stably transfected with scmbl shRNA or shRNA targeting LAMP-2 mRNA. MEF2D expression increases after starvation of the cells. However, no significant differences were observed between scmbl and LAMP-2 shRNA treated samples. GAPDH expression remained constant in all samples (#p>0.05, ***p<0.001). (d) Immunoblot and respective quantification from N2a cells showing lack of changes in macroautophagy upon knockdown of LAMP-2 (p-PRAS40 refers to phosphorylated-PRAS40; actin was used to control loading). (e) Immunoblots and respective densitometric quantification of lysates from N2a cells either untreated or starved for 3 hours to induce macroautophagy. [file 40478_2014_182_MOESM7_ESM.png]
