# Supplementary material for: LAMP-2 deficiency leads to hippocampal dysfunction but normal clearance of neuronal substrates of chaperone-mediated autophagy in a mouse model for Danon disease
Source: Acta Neuropathol Commun. 2015 Jan 31;3:6. doi: 10.1186/s40478-014-0182-y (PMC4359523; doi:10.1186/s40478-014-0182-y)
Supplement: Additional file 5: — Absence of carbohydrate storage or perturbed autophagy in LAMP-2-deficient brain. (a) Representative histological sections from LAMP-2-deficient (LAMP-2-/y) mice and their wild-type (WT) littermates stained with Periodic-Acid-Schiff (PAS). Sections were costained with the nuclear stain haematoxylin. (b) Electron micrograph showing normal morphology within the subiculum of a WT animal. (c) Immunoblots and respective densitometric quantification of hippocampal lysates. [file 40478_2014_182_MOESM5_ESM.pdf]

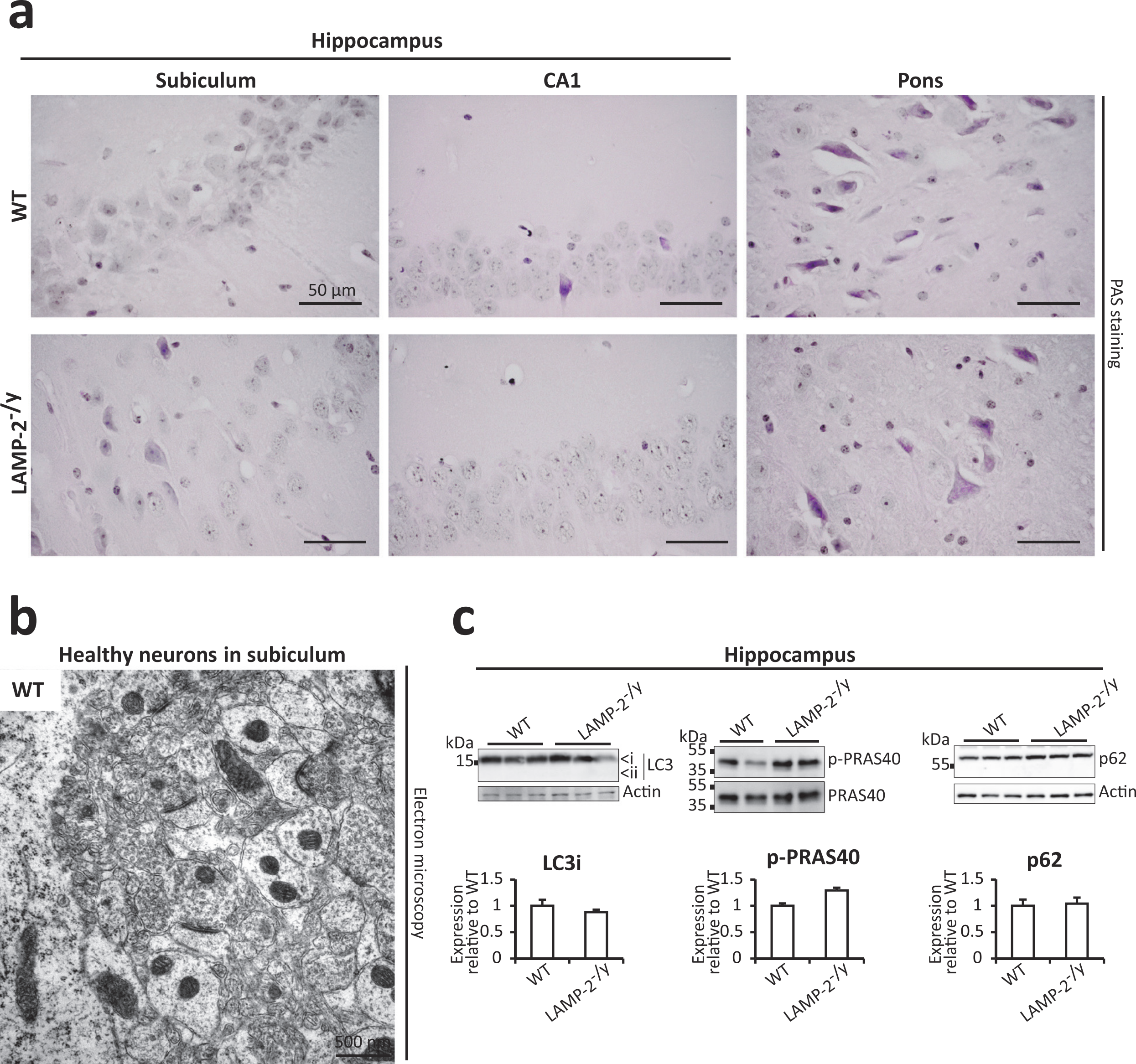

a
Hippocampus
Subiculum
CA1
Pons
WT
LAMP-2⁻/y
50 µm
PAS staining
b
Healthy neurons in subiculum
WT
500 nm
Electron microscopy
c
Hippocampus
WT
LAMP-2⁻/y
kDa
15
<i
<ii
LC3
Actin
55
35
p-PRAS40
PRAS40
p62
LC3i
p-PRAS40
p62
Expression relative to WT
1.5
1
0.5
0
